# Obesity and Diabetes in New York City, 2002 and 2004

**Published:** 2008-03-15

**Authors:** Gretchen Van Wye, Bonnie D Kerker, Donna Eisenhower, Lorna Thorpe, Shadi Chamany, Thomas Matte, Thomas R Frieden

**Affiliations:** New York City Department of Health and Mental Hygiene, Division of Health Promotion and Disease Prevention; Division of Epidemiology, New York City Department of Health and Mental Hygiene, New York, New York; Division of Epidemiology, New York City Department of Health and Mental Hygiene, New York, New York; Division of Epidemiology, New York City Department of Health and Mental Hygiene, New York, New York; Division of Health Promotion and Disease Prevention, New York City Department of Health and Mental Hygiene, New York, New York; Division of Health Promotion and Disease Prevention, New York City Department of Health and Mental Hygiene, New York, New York; Commissioner, New York City Department of Health and Mental Hygiene, New York, New York

## Abstract

**Introduction:**

Obesity and diabetes have increased rapidly nationwide, yet reliable information on these disease trends in local urban settings is unavailable. We undertook this study to characterize trends in obesity and diagnosed diabetes from 2002 to 2004 among white, black, and Hispanic adult residents of New York City.

**Methods:**

We used data from the Community Health Survey, an annual random-digit–dial telephone survey of approximately 10,000 New York City adults aged 18 years or older, and from the Behavioral Risk Factor Surveillance System, a similar nationwide survey. Main outcome measures were body mass index (BMI), calculated from self-reported height and weight, and self-reported diabetes.

**Results:**

In 2 years, the prevalence of obesity increased 17% in New York City, from 19.5% in 2002 to 22.8% in 2004 (*P* < .0001). The prevalence of diagnosed diabetes also increased 17%, from 8.1% in 2002 to 9.5% in 2004 (*P* < .01). Nationally, the prevalence of obesity increased by 6% during this same time period (*P* < .05), and diabetes prevalence did not increase significantly. The median BMI among white adults in New York City was 25.1 kg/m^2^, significantly lower than among Hispanics (26.4 kg/m^2^) and blacks (26.6 kg/m^2^, *P* < .05). The prevalence of diabetes increased across all BMI categories.

**Discussion:**

The rapid increase in obesity and diabetes in New York City suggests the severity of these twin epidemics and the importance of collecting and analyzing local data for local programming and policy making.

## Introduction

The prevalence of obesity and diabetes is increasing rapidly in the United States (1-5). Between 1990 and 2001, the Behavioral Risk Factor Surveillance System (BRFSS) documented that the prevalence of self-reported obesity increased from 1 in 10 American adults (11%) to 1 in five (21%), and diagnoses of diabetes increased from 5% to 8% (6). The increasing prevalence of these conditions is placing an additional burden on the health care system because both are associated with poor health outcomes (7). Diabetes, for example, increases the risk of myocardial infarction, congestive heart failure, stroke, retinopathy, neuropathy, nephropathy, and death (8). Obesity is a major modifiable risk factor for type 2 diabetes and is also independently associated with many other adverse health outcomes (8).

Although national trends are well documented, a limited body of data suggests that conditions vary locally, depending on sociodemographic and geographic features, such as poverty levels, racial/ethnic makeup, the size or types of immigration populations, and land-use patterns (9-16). Specifically, black and Hispanic adults have been shown to have high rates of obesity and diabetes (1), and prevalence of both conditions correlates strongly with poverty (13,14). Immigrant populations have lower average rates of obesity and diabetes (15,16), and rates of obesity are higher in rural settings and in areas marked by urban sprawl (10,12), such as those found in the southern and midwestern United States (11).

New York City is an urban environment with little developmental sprawl (17) and a large immigrant population that accounts for approximately 40% of the total population (18). These two factors would suggest lower rates of obesity and diabetes. However, the city also has poverty levels substantially higher than the national average (19) and a high concentration of black and Hispanic residents. According to national data, rates of obesity and diabetes have been rising in these two population groups (20). More than 20% of adult New Yorkers live at or below the federal poverty level, compared with 12% nationwide (19), and 48% of the city's population is black or Hispanic, compared with 24% nationally (19,21). Local estimates of obesity and diabetes for New York City are needed to understand disease burden, to monitor trends over time, and to target local prevention and control efforts.

In 2002, the Community Health Survey (CHS) was initiated to characterize and monitor the health of adults in New York City. The CHS is an annual, population-based telephone survey of approximately 10,000 randomly selected adults that provides prevalence estimates of the health of New Yorkers. In this article, we examine data from the 2002 and 2004 CHS to identify and characterize changes in body mass index (BMI) distribution among the city's adults. In addition, we examine comparable national data from the BRFSS to assess how trends in obesity and diabetes in New York City compare with national patterns.

## Methods

### The Community Health Survey 

The Community Health Survey is an annual, cross-sectional, neighborhood-stratified, random-digit–dial telephone survey that the New York City Department of Health and Mental Hygiene conducts (22). It is based on the BRFSS survey of the Centers for Disease Control and Prevention (CDC). Using a computer-assisted telephone interviewing system, the CHS randomly samples approximately 10,000 noninstitutionalized adults aged 18 years or older to obtain citywide and neighborhood-level estimates of a number of health behaviors, health care access indicators, and health conditions. Neighborhood designations are determined by a zip code–based classification system developed and used by the United Hospital Fund (23). When contact is made with a household, one adult is selected randomly to complete an interview. Interviews are conducted in the interviewee's native language. In the 2002 CHS, which was conducted from May 2002 to July 2002, 9674 interviews were completed, representing a cooperation rate of 64% (percentage of contacted adults who agreed to participate) (24). For the 2004 CHS, conducted from May 2004 to February 2005, 9585 people were interviewed, with a cooperation rate of 59%. A comparison of these samples to the U.S. Census 2000 population of New York City adults is presented in Table 1. The table shows that the CHS is representative of the adult population of New York City. Our analysis was restricted to adults who identified themselves as non-Hispanic white, non-Hispanic black, or Hispanic because the number of adults of Asian and other ethnicities was small and because the BMI guidelines for obesity may not be an appropriate measure of obesity among Asians (25). The final sample sizes used in these analyses were 8943 in 2002 and 8571 in 2004.

### The Behavioral Risk Factor Surveillance System 

The BRFSS is a cross-sectional, random-digit–dial telephone survey that is stratified by state or territory. Local health departments conduct the survey, in which data from all states and territories are pooled, in collaboration with CDC (26,27). In the 2002 BRFSS the median cooperation rate across all states was 74.3% (28). For the 2004 BRFSS the median cooperation rate across all states was 76.7% (29). After restricting the data sets to include only non-Hispanic whites, non-Hispanic blacks, and Hispanics, the final 2002 BRFSS sample size was 229,848 and the 2004 BRFSS sample size was 286,738.

### Measurements 

In both the CHS and BRFSS for 2002 and 2004, self-reported height and weight were assessed by asking, "About how tall are you without shoes?" and "About how much do you weigh without shoes?"  Obesity status was determined using the BMI, calculated by weight in kilograms divided by height in meters squared, and classified according to World Health Organization, National Institutes of Health, and CDC guidelines (BMI <18.5 kg/m^2^ for underweight, BMI 18.5–24.9 kg/m^2^ for normal weight, BMI 25–29.9 kg/m^2^ for overweight, and BMI ≥30 kg/m^2^ for obese) (30,31). Obesity is further categorized into three classes: BMI from 30 to 34.9 kg/m^2^ is defined as Class I obesity, BMI from 35 to 39.9 kg/m^2^ as Class II obesity, and BMI of 40 kg/m^2^ or greater as Class III, or severe, obesity (2,31). Because of small numbers (n = 1001 in 2002 and n = 689 in 2004) of adults with a BMI less than 18.5 kg/m^2^, the two lowest categories were collapsed into one (underweight/normal weight). In both the CHS and BRFSS, diabetes was assessed by asking "Have you ever been told by a doctor that you have diabetes?"  Women who reported having had gestational diabetes exclusively were not considered to have diabetes.

We obtained New York City neighborhood income levels from U.S. Census 2000 data; income level was defined as the percentage of each neighborhood living below 200% of the federal poverty level and was stratified into categories of high, medium, and low. Neighborhoods in which 45% to 90% of the population lived at or below 200% of the federal poverty level were considered low income, and neighborhoods in which less than 30% of the population lived at or below 200% of the federal poverty level were defined as middle- or high-income neighborhoods. Adults were defined as foreign born if they reported a birthplace outside of the United States, Puerto Rico, or other U.S. territories except in the Hispanic subgroup analysis, for which Hispanics were assessed as either U.S.-born, foreign-born, or born in Puerto Rico. The Hispanic subgroup analysis was performed because New York City Hispanic subgroups may be different from subgroups in other parts of the United States because of different birth and migration patterns.

### Analysis 

Each record in the CHS was assigned a primary weight for the probability of selection (i.e., number of adults in each household, number of residential telephone lines) and a post-stratification weight in order to adjust the sample estimates to the composition of each neighborhood in age, race/ethnicity, and sex (32). Similarly, each record in the BRFSS was assigned both a primary weight and a post-stratification weight in order to adjust the sample estimates to the composition of the states and country in age, race/ethnicity, and sex (33). We calculated the BMI distribution and the prevalence of obesity and diabetes in New York City and the United States in 2002 and 2004, stratified by demographic subgroup. Then, we examined the change in prevalence of obesity and diabetes from 2002 to 2004 in order to assess whether either had increased over time. Finally, we compared trends in New York City and the United States. To compare the prevalence of obesity and diabetes across years and demographic subgroups in the CHS and BRFSS, *t* tests were calculated; BMI quartiles in New York City in 2002 and 2004 were compared using 95% confidence intervals. In addition, logistic regression was performed to assess the independence of the within-year effects of the demographic variables on obesity and diabetes in New York City. Models were built in a forward stepwise manner, and variables were retained in the model if they were statistically significant at α = .05. National estimates were compared with those of New York City using 95% confidence intervals. SAS and SAS-callable SUDAAN 9 (Research Triangle Institute, Research Triangle Park, North Carolina) were used to perform these analyses.

## Results

### Obesity 

**Figure 1:**
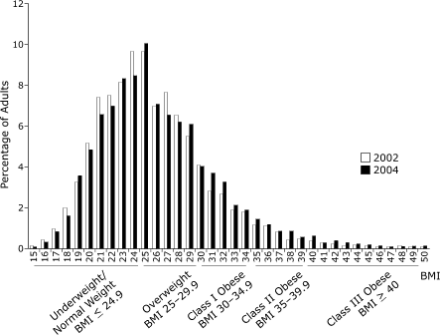
2002 and 2004 BMI distributions among white, black, and Hispanic adults in New York City.

The age-adjusted prevalence of obesity in New York City increased from 19.5% in 2002 to 22.8% in 2004 (*P* < .001), representing an additional 173,500 obese adults or a 17.0% increase in 2 years (Table 2). Nationally, the overall prevalence of obesity increased by 6% during the same time period (from 21.3% to 22.7%, *P* < .001) (Table 2). The 2004 increase in obesity prevalence among adult New Yorkers was the result of a shift in the entire BMI distribution to higher values (Figure 1), yielding a smaller proportion of adults in the underweight/normal weight category (*P* < .001) and a larger proportion in the obese category (*P* < .001), compared with 2002. The proportion of adults in the overweight category did not change significantly from 2002 to 2004 (from 36.1% to 35.8%, *P* = .78); however, the total population that was either overweight or obese increased from 55.6% to 58.7% (*P* < .001). Although the upward shifts of the 25th and 50th (median) percentiles of BMI were not significant (*P* = .05), the larger shift of the 75th percentile (by .6 BMI-unit) was significant at *P* < .05.

**Table:** 

During the 2 years under study, the prevalence of obesity rose in all sociodemographic groups, but increases were statistically significant among only certain subgroups. Significant increases occurred in both sex and nativity subgroups, both U.S.-born and foreign-born, as well as among adults aged 25 to 44 years and adults aged 65 or older. Obesity levels also increased significantly among whites (14.3%–17.1%, *P* < .01) and among Hispanics (22.9%–26.2%, *P *<.05), but not among blacks (25.7%–29.0%, *P* = .052). When we examined the prevalence of obesity among subgroups of Hispanics, we observed significant increases among both U.S.-born (28.0%–35.8%, *P* < .05) and foreign-born (16.3%–23.1%, *P* < .05), but not among adults born in Puerto Rico (28.4%–29.7%, *P* = .78), despite overall high levels in the Hispanic group. At the national level, there were also significant increases in obesity in male and female subgroups (*P* < .05). In contrast to the CHS data, national obesity rates increased among all age groups except among those aged 65 or older. The largest increase in obesity occurred in those aged 18 to 24 years, for whom obesity increased by 17% (12%–14%, *P* < .05). Nationally, the only racial/ethnic group with a significant increase in obesity was whites (*P* < .01). Because the subgroups of Hispanics who live in New York City may be different from the subgroups that live in the United States overall, we performed a multivariate analysis of Hispanic New Yorkers to determine if the levels of obesity among subgroups held when accounting for other sociodemographic variables. In this analysis, the independent effect of the Hispanic subgroup was consistent with previous results (i.e., the association between Puerto Rican- and U.S.-born Hispanics and obesity held). In fact, all sociodemographic variables had a significant independent effect on obesity (*P* < .05). Because nativity data are not collected by the national BRFSS, a national comparison was not possible.

**Figure 2:**
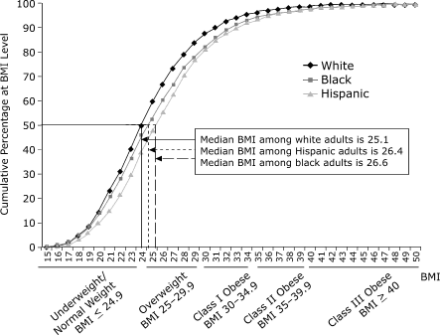
Cumulative percentage of New York City race/ethnicity-specific population groups at or below BMI level, 2002–2004, pooled.

Figure 2 illustrates the cumulative percentage of adult New Yorkers at or below each BMI point on the basis of pooled BMI distributions by race/ethnicity for 2002 and 2004. The distribution illustrates that the weight distribution among whites was concentrated at lower BMI levels compared with the distribution among blacks and Hispanics; the median BMI among white adults was 25.1 kg/m^2^, significantly lower than among Hispanics (26.4 kg/m^2^, *P* < .05) and blacks (26.6 kg/m^2^, *P* < .05). In addition, whites were more likely than blacks and Hispanics to be in the underweight/normal weight category and less likely to be in the overweight and obese categories (*P* < .05).

**Table:** 

### Diabetes 

In New York City, the prevalence of diabetes increased by 17% from 2002 to 2004 (8.1%–9.5%, *P* < .01) (Table 3), with an estimated 73,600 more adults reporting that they were diagnosed with diabetes in 2004. Diabetes increased across all racial and ethnic groups. Significant increases in diabetes occurred among several other demographic subgroups: prevalence increased by 24% among men (8.1%–10.0%, *P* < .05), by 23% among adults aged 65 and older (19.0%–23.4%, *P* < .05), and by 24% among whites (5.1%–6.3%, *P* < .05). Nationally, there was no statistically significant increase in the overall prevalence of diabetes from 2002 to 2004 (6.9%–7.1%, *P* = .48), although there was a significant increase in the prevalence of diabetes among men (7.2%–7.7%, *P* < .05) (Table 3). In New York City, the prevalence of diabetes increased significantly among individuals who were underweight/normal weight (3.7%–5.2%, *P* < .05) (Table 4), although nonsignificant increases were observed across the other BMI categories (overweight and obese).

## Discussion

This study documents a rapid increase in obesity and diabetes within a 2-year time period among adults in New York City, larger than that observed in the United States overall. The 17% increase in prevalence of self-reported obesity that occurred from 2002 to 2004 corresponds to an additional 173,500 obese adults, and the 17% increase in diabetes prevalence, corresponds to approximately 73,600 additional adults reporting a diagnosis of diabetes. As of 2004, nearly 1 in 4 adults in New York City were obese, and 1 in 10 had diagnosed diabetes. The rapid rise of obesity in the city has brought prevalence to a level comparable to the national average.

The increase in obesity among adult New Yorkers corresponds to an average weight gain of 2 pounds per person between 2002 and 2004, indicating a total citywide weight gain of more than 10 million pounds, with the largest increases occurring at the higher end of the weight spectrum (i.e., 75th percentile). Between 2002 and 2004, the change in obesity was different in New York City than in the United States. For instance, obesity increased among both whites (20%, *P* < .05) and Hispanics (14%, *P* < .05), but national increases were significant only among whites (7%, *P* < .05). Thus, the 2-year rate of increase in obesity was higher than the national increase in the two largest racial/ethnic subpopulations that together comprise nearly two-thirds of the total population of New York City: 38% of adults are white, 23% are black, 25% are Hispanic, and 14% are Asian/Pacific Islander or of another racial/ethnic group (19). The increase in obesity was also considerable among older New Yorkers (28%), whereas estimates of obesity among older adults in the United States overall were stable over time. Finally, there was a dramatic (33%) increase in the prevalence of obesity among foreign-born New Yorkers from 2002 to 2004.

Although some characteristics of the New York City population, such as its racial/ethnic profile and its higher level of poverty, suggest that rates of obesity should be higher than the national average, in 2004 its obesity rate was comparable to national levels, not higher. Although we documented an income-associated gradient for obesity among adult New Yorkers as well as higher rates among Hispanic and black residents than rates among whites, the race-specific obesity levels among whites and blacks in New York City were comparable to national levels. Because of differences in methods of measuring income in the CHS and BRFSS, a direct comparison of obesity levels by poverty level was not possible for this study. Still, the obesity estimates for New York City compared with those for the United States overall, despite the high proportions of black, Hispanic, and poor adults in the city, suggest that other factors have a protective effect on local obesity levels.

One factor that may attenuate obesity levels in New York City is nativity. Research has demonstrated an inverse association between foreign-born status and obesity (16), and the lower prevalence of obesity that we observed among foreign-born adults in the city is consistent with this finding. In New York City, foreign-born adults comprise 44% of the adult population (19), compared with only 13% of the adult population of the United States (21), and the significantly lower obesity levels among foreign-born residents influenced race-specific and overall obesity levels. We were unable to make a direct comparison of obesity levels by country of birth in this study because BRFSS does not collect data on nativity for foreign-born U.S. residents.

Another possible explanation for why the 2004 prevalence of obesity in New York City is lower than its sociodemographic makeup might suggest is urban design. With neighborhoods that are limited to defined geographic boundaries, largely completed by the 1950s and 1960s, New York City is a generally walkable environment, characterized by mixed land use, and connected both internally and externally by rail transportation systems (17), making it relatively small, with both retail and residential destinations easily accessible by public transportation and by foot. However, given the stability of the city's built environment, the rapid rise in obesity suggests that other factors are driving the increase over time. Because the urban design of New York City may be considerably different from that of other parts of the country, particularly in the southern and midwestern United States, future research should investigate the impact of the built environment on obesity and diabetes in the context of other factors, including race, ethnicity, poverty, and sociocultural factors that affect obesity and diabetes.

The prevalence of diabetes increased significantly in New York City from 2002 to 2004, whereas it remained constant nationally during that time. This increase was significant among men, older adults, whites, and those living in higher income neighborhoods. Increases were also significant among both U.S.-born and foreign-born adults, but were more marked among foreign-born adults (26% vs 15% increase in 2 years). These findings suggest that more adult New Yorkers, particularly those in the wealthier segments of the population, are developing diabetes. The higher 2004 prevalence may also reflect recent increases in diabetes screening in some subpopulations of the city.

In contrast to our findings on obesity, we found that the prevalence of diabetes in New York City surpassed the national prevalence in 2004 (9.5% vs 7.1%, *P* < .05). The higher prevalence largely reflects the high rates of this disease among poorer residents and among black and Hispanic adults, suggesting that fewer local protective factors may exist for diabetes than for obesity. Indeed, the prevalence of diabetes among Hispanic New Yorkers was higher than that of Hispanics in the United States overall (13.1% vs 9.8%, *P* < .05). Prevalence of the disease among people aged 65 or older was also higher than in the United States overall for that age group (23.2% vs 16.6%, *P* < .05).

In the future, the prevalence of obesity and diabetes in both New York City and in the United States will be affected by growth in the populations that experienced the largest increases in these conditions between 2002 and 2004, specifically older adults, Hispanics, and the foreign-born (34). Adults aged 65 or older currently comprise about 12% of the population, both in New York City and in the United States (19,21). This age group is projected to grow more rapidly than any other within the next several decades, partly because of the aging of the baby boom generation (34). Similarly, Hispanics are expected to comprise 23% of the U.S. population by the year 2050, and immigration is projected as a primary driver of overall population growth (34). Understanding and responding to the impact of these changes in the population groups of New York City and the United States will continue to require local and national data.

### Limitations and strengths 

Limitations of this analysis include those related to self-reported data. Specifically, because data from the CHS are self-reported, estimates of obesity are likely to be low; people typically overstate their height and understate their weight (35). Similarly, our estimates of diabetes are likely to be low because not all adults with diabetes will recall their diabetes status during an interview and because diabetes is often undiagnosed; about 30% of adults with diabetes do not know they have it (36). However, because the questions were identical in 2002 and 2004 and because these samples are highly comparable, underreporting is not expected to have varied between years and would thus not affect our analysis. Additional limitations of the study include its cross-sectional design, which limits our ability to assess temporality or track incident conditions. Institutionalized adults and those without telephones were not represented in the sampling frame, limiting the generalizability of our findings. In addition, bias may have been introduced as a result of perceived pressure to provide socially desirable answers; however, the anonymous nature of the survey may have limited this effect (22,37). Because local and national data were collected with the same survey method, these limitations should not affect our comparisons. The large difference in the size of the BRFSS and CHS samples is an additional limitation because larger population samples, such as the BRFSS sample, are more likely to yield statistically significant results than smaller samples, such as the CHS. Therefore, all else being equal, the BRFSS would yield more statistically significant differences than the CHS. However, there were actually more statistically significant differences between years in New York City than in the United States. Finally, this trend analysis is limited to only 2 years of data. Additional research examining data from the CHS and BRFSS should be ongoing in the future to assess longer-term trends. On the other hand, the smaller CHS sample means less precise estimation of trends compared to BRFSS. Strengths of the CHS include representativeness because it is conducted in multiple languages and its data characterizes the adult New York City population.

### Conclusions

From 2002 to 2004, an additional 173,500 adult New Yorkers became obese, and an additional 73,600 were diagnosed with diabetes. Increases in obesity and diabetes were largest among some of the most rapidly growing subgroups in New York City and the United States, suggesting that the health impact and burden to the health care system related to these conditions may accelerate in coming years. Differences in obesity and diabetes between New York City and the United States underscore the need for local data. Understanding trends is important for local programming and policy making. Without immediate action, both New York City and the United States as a whole will experience increasingly urgent and damaging epidemics.

## Figures and Tables

**Table 1:** Age and Race/Ethnicity Distributions, U.S. Census 2000 and Community Health Survey 2002 and 2004

Race/Ethnicity, by Age (y)	Percentage of New York City Adults in Age-Race Category a		

	U.S. Census 2000	CHS 2002	CHS 2004
**White**			
18-24	3.8	3.0	2.1
25-44	14.8	16.0	13.0
45-64	11.3	13.1	13.6
≥65	8.8	10.0	11.3
**Black**			
18-24	3.2	2.6	2.3
25-44	10.0	10.8	10.1
45-64	6.7	7.3	7.6
≥65	3.1	3.6	3.6
**Hispanic**			
18-24	4.3	3.9	3.0
25-44	11.8	12.7	12.5
45-64	6.3	6.8	7.5
≥65	2.3	2.6	2.6
**Asian/Pacific Islander**			
18-24	1.4	.9	.9
25-44	5.0	3.2	3.6
45-64	2.8	1.2	2.0
≥65	1.0	.3	.9

a Percentages do not total 100 because the table does not include the "Other" race/ethnicity category.

**Table 2:** Age-Adjusted Prevalence of Obesity in New York City and the United States, Community Health Survey and Behavioral and Risk Factor Surveillance System, 2002 and 2004^a^

Demographic Characteristics	New York City		United States	

	2002 % (SE)	2004 % (SE)	2002 % (SE)	2004 % (SE)
**Totalb, c**	19.5 (.55)	22.8 (.57)	21.3 (.16)	22.7 (.16)
**Sex**				
Menb, c	17.7 (.84)	21.0 (.87)	22.3 (.24)	23.8 (.25)
Women	20.9 (.73)	24.2 (.74)	20.4 (.19)	21.7 (.19)
**Age group**				
18-24^c^	10.4 (1.32)	13.7 (1.67)	12.0 (.44)	14.0 (.50)
25-44b, c	16.4 (.75)	20.6 (.87)	21.4 (.26)	23.5 (.26)
45-64	27.2 (1.21)	28.0 (1.1)	26.2 (.29)	27.3 (.27)
≥65b, c	20.2 (1.35)	25.9 (1.3)	19.7 (.32)	19.4 (.30)
**Race/ethnicity**				
Whiteb, c	14.3 (.73)	17.1 (.78)	19.6 (.15)	21 (.16)
Black	25.7 (1.16)	29.0 (1.20)	32.2 (.58)	32.7 (.53)
Hispanicb, c	22.9 (1.15)	26.2 (1.20)	22.9 (.66)	24.3 (.60)
**Nativity**				
U.S.-bornb, c	20.7 (.65)	23.2 (.70)	NA	NA
Foreign-bornb, c, d	16.8 (1.01)	22.4 (.99)	NA	NA
**Neighborhood income**				
High	15.5 (0.87)	17.7 (.93)	NA	NA
Medium^b^	19.2 (1.01)	24.5 (1.0)	NA	NA
Low	23.8 (.93)	26.0 (1.0)	NA	NA

NA indicates data not available.

a Age-specific estimates are not age-adjusted.

b Change in obesity in New York City from 2002 to 2004 statistically significant at *P* < .05.

c Change in obesity in United States from 2002 to 2004 statistically significant at *P* < .05.

d Foreign-born includes individuals born in Puerto Rico and other U.S. territories.

**Table 3:** Age-Adjusted Prevalence of Diabetes in New York City and the United States, Community Health Survey and Behavioral and Risk Factor Surveillance System, 2002 and 2004

Demographic Characteristics	New York City		United States	

	2002 % (SE)	2004 % (SE)	2002 % (SE)	2004 % (SE)
**Total^a^**	8.1 (.34)	9.5 (.36)	6.9 (.09)	7.1 (.09)
**Sex**				
Menb, c	8.1 (.55)	10.0 (.60)	7.2 (.15)	7.7 (.15)
Women	8.0 (.44)	9.2 (.43)	6.7 (.12)	6.5 (.10)
**Age group**				
18-24	0.6 (.26)	1.2 (.42)	0.88 (.14)	0.75 (.09)
25-44^b^	3.0 (.35)	3.4 (.37)	2.6 (.10)	2.8 (.11)
45-64	11.8 (.78)	13.5 (.80)	9.8 (.20)	10.1 (.19)
≥65^b^	19.0 (1.22)	23.4 (1.23)	16.6 (.33)	16.6 (.30)
**Race/ethnicity**				
White^b^	5.1 (.40)	6.3 (.42)	5.9 (.08)	6.2 (.08)
Black	10.9 (.79)	13.0 (.79)	12.4 (.40)	11.8 (.36)
Hispanic	12.3 (.86)	13.1 (.86)	10.6 (.58)	9.8 (.47)
**Nativity**				
U.S.-born^b^	8.0 (.40)	9.2 (.43)	NA	NA
Foreign-bornb, d	8.0 (.66)	10.1 (.63)	NA	NA
**Neighborhood income**				
High^b^	5.4 (.51)	7.5 (.59)	NA	NA
Medium^b^	7.8 (.60)	9.4 (.58)	NA	NA
Low	11.2 (.67)	11.9 (.69)	NA	NA

SE indicates standard error; NA, data not available

a Age-specific estimates are not age-adjusted.

b Change in diabetes in New York City from 2002 to 2004 is statistically significant at *P* < .05.

c Change in diabetes in United States from 2002 to 2004 is statistically significant at *P* < .05.

d Foreign-born includes individuals born in Puerto Rico and other U.S. territories.

**Table 4:** Age-Adjusted Prevalence of Diabetes by Weight Category, New York City and United States, 2002 and 2004

Weight Category	New York City^a^		United States	

	2002 % (SE)	2004 % (SE)	2002 % (SE)	2004 % (SE)
Underweight/normal weight	3.7 (.39)	5.2 (.47)	3.9 (.12)	3.2 (.11)
Overweight	8.1 (.60)	9.0 (.59)	5.9 (.15)	5.9 (.13)
Obese	15.4 (.99)	16.5 (.92)	13.6 (.26)	14.13 (.24)

a Change in diabetes in New York City from 2002 to 2004 is statistically significant at *P* < .05.
